# Adult lung transplantation case-volume and in-hospital and long-term mortality in Korea

**DOI:** 10.1186/s13019-019-0849-3

**Published:** 2019-01-23

**Authors:** Susie Yoon, Eun Jin Jang, Ga Hee Kim, Dal Ho Kim, Tae-Yoon Lim, Hannah Lee, Ho Geol Ryu

**Affiliations:** 1 Department of Anaesthesiology and Pain Medicine, Seoul National University College of Medicine, Seoul National University Hospital, 101 Daehak-ro, Jongno-gu, Seoul, 03080 South Korea; 2 Department of Information Statistics, Andong National University, 1375 Gyeongdong-ro, Andong-si, Gyeongsangbuk-do 36729 South Korea; 3 Department of Statistics, Kyungpook National University, 80 Daehak-ro, Buk-gu, Daegu, 41566 South Korea; 4 Department of Statistics, Kyungpook National University, 80 Daehak-ro, Buk-gu, Daegu, 41566 South Korea

**Keywords:** Case-volume, Lung transplantation, In-hospital mortality

## Abstract

**Background:**

The inverse relationship between case-volume and surgical mortality has been reported in complex surgical procedures. The aim of this study was to evaluate the effect of case-volume on mortality after lung transplantation in Korea.

**Methods:**

The National Health Insurance Service data was used to analyse all adult lung transplantations in Korea between 2007 and 2016. Institutions were categorized into low-volume (< 5 lung transplantations/year) centers or high-volume (≥ 5 lung transplantations/year) centers. Risk-adjusted in-hospital mortality and long-term survival according to case-volume was evaluated.

**Results:**

A total of 315 adult recipients underwent lung transplantation at 7 centers. The odds ratio for in-hospital mortality in low-volume centers was similar to high-volume centers (OR, 1.496; 95% CI, 0.81–2.76; *p* = 0.197). Log-rank analysis of Kaplan-Meier curves according to case-volume also did not show a difference in long-term survival between high- and low-volume centers (*p* = 0.052).

**Conclusions:**

There was no association between case-volume and in-hospital mortality after lung transplantation in Korea, although there was a tendency towards better long-term survival associated with high-volume centers.

## Background

Lung transplantation (LT) is the definitive treatment for end-stage lung disease [1]. Mortality rates after LT are high compared to other solid organ transplantations due to the complexity of the procedure and perioperative management [2]. More than 4000 patients receive LT every year worldwide and patient outcomes are steadily improving [3]. The International Society for Heart and Lung Transplantation (ISHLT) reported a median survival of six years in adult patients who underwent LT between 1990 and 2015 [3], with one-year survival improving from 72 to 84% during the past seven years [3].

The first LT in Korea was performed in 1996 and the cumulative number of LTs is approaching 400 [4]. Although the nationwide volume of LT in Korea is relatively modest compared to other solid organ transplantations in Korea and LTs elsewhere, the number of LTs performed in Korea has increased significantly in the past decade. Of note, the most common lung disease leading to LT in Korea was idiopathic pulmonary fibrosis (IPF) which comprised approximately 50% of LTs, whereas chronic obstructive pulmonary disease (COPD) is the most frequently reported cause in western countries [3, 4].

The inverse relationship between case-volume and surgical mortality has been reported in complex surgical procedures such as high-risk cancer surgery [5, 6], cardiovascular surgery [7–9], and liver transplantation [10, 11]. A similar relationship has been reported after LT [2, 12–15], but has not been shown in Korea where the majority of LTs are performed in patients with IPF or interstitial lung disease (ILD) [4]. The objective of this study was to evaluate the effect of case-volume on mortality after LT from 2007 to 2016 in Korea.

## Methods

This retrospective cohort study was approved by the institutional review board of Seoul National University Hospital (IRB #1704–016-842) and all study protocols were in accordance with guidelines and regulations approved by the ethics review committee for human studies of Seoul National University Hospital. Informed consent was waived by the institutional review board due to the retrospective nature of the study design and lack of feasibility.

The National Health Insurance Service (NHIS) governs the mandatory universal healthcare coverage system of Korea [16]. The National Health Insurance (NHI) program which provides healthcare to more than 97% of Koreans, is regulated by the NHIS [17]. Therefore, nearly all LTs performed for the entire Korean population are represented in the NHIS data [17].

All adult patients (age ≥ 19) who underwent LT between 2007 and 2016 were identified by searching and identifying the procedure code of single LT (Q8101) and double LT (Q8102). Pulmonary diseases that led to LT were extracted using ICD-10 codes; ILD (J849), IPF (J8418), COPD (J438–41, J448–9) and acute respiratory distress syndrome (J80). Comorbidities such as hypertension, diabetes mellitus, coronary artery disease, chronic kidney disease, and cerebrovascular disease were also extracted from the database. The Elixhauser comorbidity index, calculated from 30 disease entities using ICD-10 codes [18], and shown to correlate with patient outcome [19], was used as a covariate to adjust for patients’ severity of illness. Data on in-hospital mortality, long-term survival, intensive care unit (ICU) and hospital length of stay (LOS) were also extracted.

The institutional case-volume was defined as the average number of LTs performed per year between 2011 and 2016. Transplant centers were then categorized according to the case-volume; low volume centers (< 5 LTs/year) and high volume centers (≥ 5 LTs/year).

The primary outcome was in-hospital mortality as short-term mortality after LT. We also evaluated long-term survival followed for up to seven years stratified by case-volume. Other outcomes included ICU and hospital length of stay.

Data were presented as mean and standard deviation or frequency and proportion, or proportion as appropriate. In-hospital mortality after LT according to the case-volume was evaluated using logistic regression model after adjusting for age, sex, transplantation period, and Elixhauser comorbidity index. The results from the logistic regression were presented as odds ratio (OR) and 95% confidence interval (CI). Survival after LT was compared using the Cox proportional hazard model after adjusting for age, sex, and Elixhauser comorbidity index. The results from the Cox regression were summarized as hazard ratio (HR) and 95% CI. In addition, Kaplan-Meier survival curves were generated and log-rank test was performed for comparison. ICU length of stay and hospital length of stay according to the case-volume were analysed using the analysis of variance method.

All analyses were performed using SAS 9.4 (SAS Institute, Cary, NC). A *p*-value < 0.05 was regarded as statistically significant.

## Results

A total of 315 adult patients underwent LT at seven centers in Korea between 2007 and 2016. The median case-volume was 4.0 (1–25.33) LTs per year. Baseline patient and center characteristics are summarized in Table 1. The most common pulmonary disease leading to LT was ILD followed by IPF. Both low- and high-volume centers showed a similar distribution of lung disease (Table 1).

**Table 1:** Baseline characteristics of lung transplantation recipients according to case-volume

Characteristics	Total	Low volume centers^a^ (< 5 LTs/year)	High volume centers^a^ (≥ 5 LTs/year)	*P* value
Number of recipients	315	71	244	
Number of centers	7	4	3	
Sex				0.897
Male	184 (58.4)	41 (57.7)	143 (58.6)	
Female	131 (41.6)	30 (42.3)	101 (41.4)	
Age				0.769
19 ≤ age < 50	118 (37.5)	24 (33.8)	94 (38.5)	0.769
50 ≤ age < 60	96 (30.5)	23 (32.4)	73 (29.9)	
60 ≤ age	101 (32.1)	24 (33.8)	77 (31.6)	
Diagnosis				0.286
Interstitial lung disease	121 (38.4)	25 (35.2)	96 (39.3)	
Idiopathic pulmonary fibrosis	95 (30.2)	20 (28.2)	75 (30.7)	
Chronic Obstructive Pulmonary Disease	67 (21.3)	18 (25.4)	49 (20.1)	
Acute Respiratory Distress Syndrome	8 (2.5)	4 (5.6)	4 (1.6)	
Others	24 (7.6)	4 (5.6)	20 (8.2)	
Comorbidities				
Hypertension	123 (39.0)	29 (39.4)	95 (38.9)	0.939
Diabetes mellitus	96 (30.5)	18 (25.4)	78 (32.0)	0.287
Coronary artery disease	45 (14.3)	16 (22.5)	29 (11.9)	0.024
Chronic kidney disease	21 (6.7)		21 (8.6)	0.006
Cerebrovascular disease	16 (5.1)	2 (2.8)	14 (5.7)	0.539
Elixhauser comorbidity index	14.6 ± 9.81	16.59 ± 9.62	14.02 ± 9.81	0.052

Data are presented as n (%) or mean ± SD

*LT* lung transplantation

^a^Average number of lung transplantations from 2011 to 2016

**Fig. 1:**
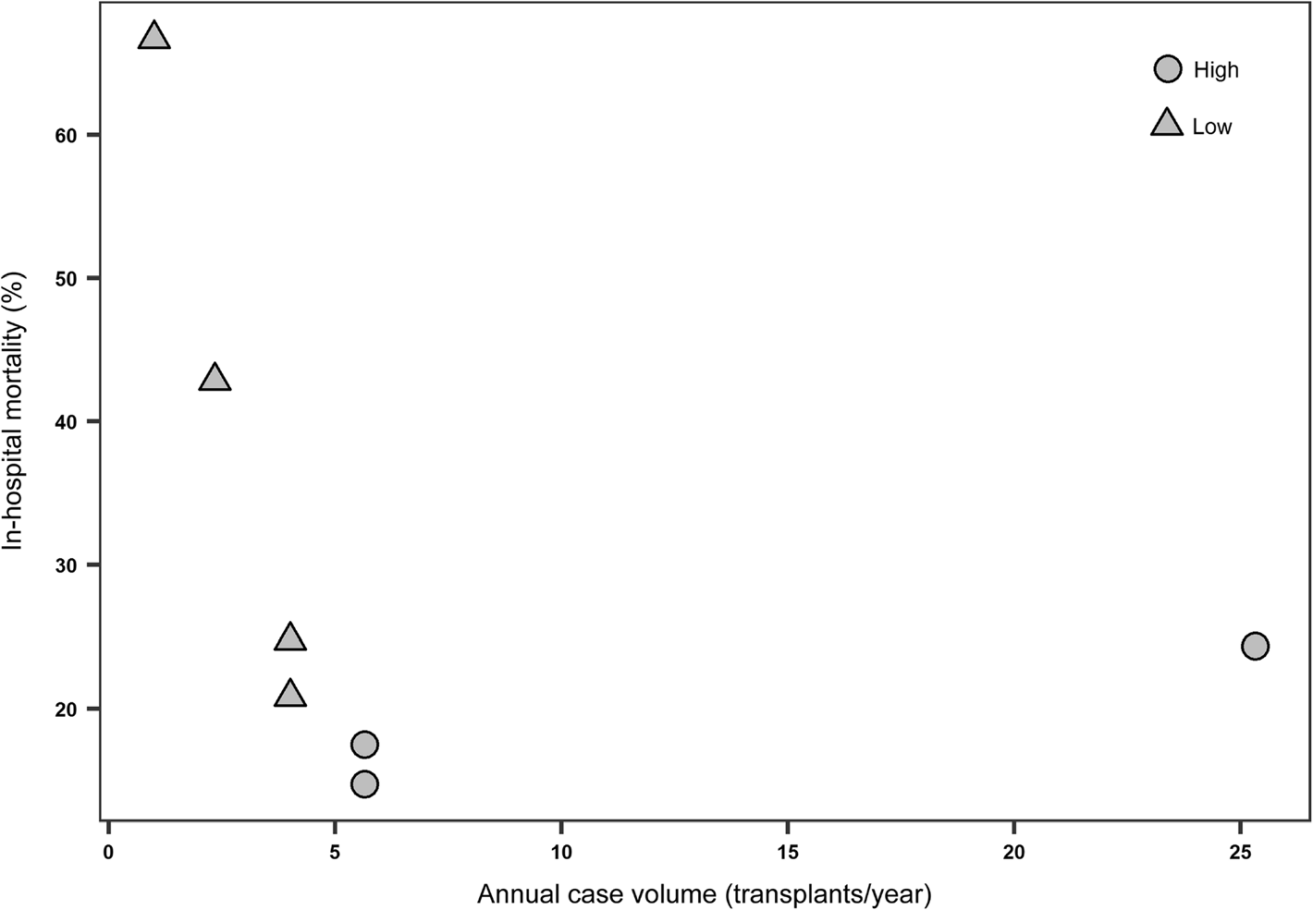
Relation between the number of cumulative cases and in-hospital mortality after lung transplantation

The overall in-hospital mortality after LT was 25.7% (81/315). In-hospital mortality after LT in low- and high-volume centers were 32.4% (23/71) and 23.8% (58/244), respectively (Table 2). The relationship between the annual number of LT cases and in-hospital mortality after LT is shown in Fig. 1.

**Table 2:** Logistic regression for in-hospital mortality after lung transplantation

	In-hospital mortality, n (%)	Unadjusted		Adjusted	
		OR (95% CI)	*P* value	OR (95% CI)	*P* value
Case-volume					
Low (< 5 LTs/year)	23 (32.4)	1.537 (0.862, 2.739)	0.145	1.496 (0.811, 2.758)	0.197
High (≥ 5 LTs/year)	58 (23.8)			Reference	
Age					
19 ≤ age < 50	22 (18.6)			Reference	
50 ≤ age < 60	24 (25.0)	1.455 (0.756, 2.798)	0.26	1.867 (0.902, 3.867)	0.093
60 ≤ age	35 (34.7)	2.314 (1.247, 4.295)	0.007	3.533 (1.671, 7.47)	< 0.001
Sex					
Male	50 (27.2)			Reference	
Female	31 (23.7)	0.831 (0.495, 1.394)	0.48	0.917 (0.517, 1.628)	0.767
Diagnosis					
ILD	27 (22.3)			Reference	
IPF	25 (26.3)	1.243 (0.665, 2.325)	0.49	1.244 (0.639, 2.423)	0.52
COPD	17 (25.4)	1.184 (0.59, 2.377)	0.63	1.508 (0.704, 3.232)	0.291
ARDS	3 (37.5)	2.089 (0.469, 9.305)	0.33	2.835 (0.587, 13.68)	0.195
Others	9 (37.5)	2.089 (0.824, 5.298)	0.12	2.33 (0.819, 6.628)	0.113
Elixhauser comorbidity index		0.998 (0.972, 1.024)	0.87	0.995 (0.967, 1.023)	0.706
Transplantation period					
2007–2010	14 (45.2)	3.047 (1.384, 6.706)	0.005	3.949 (1.618, 9.638)	0.003
2011–2013	27 (28.1)	1.448 (0.822, 2.549)	0.19	1.729 (0.948, 3.153)	0.074
2014–2016	40 (21.3)			Reference	

*OR* odds ratio, *IPF* idiopathic pulmonary fibrosis, *ILD* interstitial lung disease, *COPD* chronic obstructive pulmonary disease, *ARDS*, acute respiratory distress syndrome

After adjusting for age, sex, transplantation period, and Elixhauser comorbidity index, the odds ratio for in-hospital mortality in low-volume centers was similar to high-volume centers (OR, 1.496; 95% CI, 0.81–2.76; *p* = 0.197; Table 2). Patients who were 60 years or older showed higher risk-adjusted in-hospital mortality compared to patients under 50 years (OR, 3.53; 95% CI, 1.67–7.47; *p* < 0.001; Table 2). Adjusted in-hospital mortality after LT during the most recent 3 years (2014–16) was significantly lower than the first 4 years of the study period (2007–10; OR, 3.94; 95% CI, 1.61–9.63; *p* = 0.002; Table 2). There was no association between baseline pulmonary diagnosis and in-hospital mortality after lung transplantation.

**Fig. 2:**
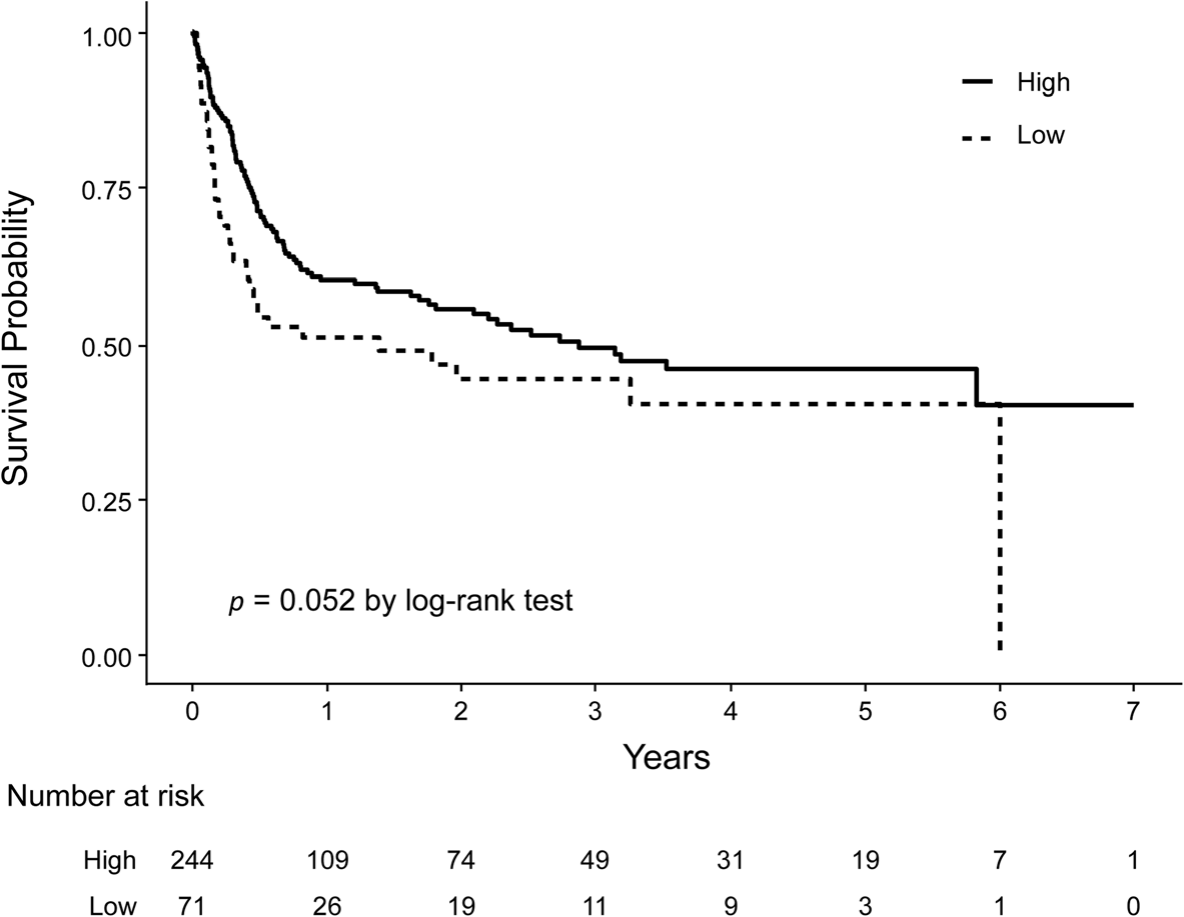
Kaplan-Meier estimates of survival stratified by case-volume

In the Cox proportional hazard model for patient survival after LT, patients who were between 50 and 60 or more than 60 years were associated with a higher mortality compared to patients who were less than 50 years old (HR, 1.75; 95% CI, 1.12–2.75; *p* = 0.01 and HR, 2.87; 95% CI, 1.82–4.53; *p* < 0.001, respectively). Furthermore, COPD patients showed worse survival after LT compared to patients with ILD (HR, 1.7; 95% CI, 1.06–2.73; *p* = 0.025). However, the impact of case-volume was not significant (HR, 1.37; 95% CI, 0.94–1.99; *p* = 0.09) (Table 3). Kaplan-Meier analysis also failed to show a statistically significant difference in long-term survival between the case-volumes (*p* = 0.052 by log-rank test; Fig. 2).

**Table 3:** Cox proportional hazard model for patient survival after lung transplantation

	Mortality, n (%)	Unadjusted			Adjusted	
		HR (95% CI)		*P* value	HR (95% CI)	*P* value
Case-volume						
Low (< 5 LTs/year)	39 (54.9)	1.435 (0.994, 2.072)		0.054	1.373 (0.943, 1.999)	0.098
High (≥ 5 LTs/year)	106 (43.4)				Reference	
Age						
19 ≤ age < 50	41 (34.7)				Reference	
50 ≤ age < 60	43 (44.8)	1.546 (1.003, 2.376)		0.046	1.756 (1.12, 2.755)	0.014
60 ≤ age	61 (60.4)	2.398 (1.606, 3.580)		< 0.001	2.873 (1.82, 4.536)	< 0.001
Sex						
Male	89 (48.4)				Reference	
Female	56 (42.7)	0.768 (0.549, 1.074)		0.12	0.864 (0.604, 1.237)	0.426
Diagnosis						
ILD	47 (38.8)				Reference	
IPF	44 (46.3)	1.312 (0.869, 1.981)		0.19	1.11 (0.73, 1.686)	0.626
COPD	33 (49.3)	1.264 (0.809, 1.974)		0.30	1.709 (1.069, 2.733)	0.025
ARDS	4 (50.0)	0.488 (0.536, 4.132)		0.44	1.329 (0.475, 3.72)	0.588
Others	17 (70.8)	1.963 (1.125, 3.426)		0.017	2.76 (1.542, 4.941)	< 0.001
Elixhauser comorbidity index		1.004 (0.987, 1.020)		0.65	1 (0.983, 1.017)	0.991

*HR* hazard ratio, *IPF* idiopathic pulmonary fibrosis, *ILD* interstitial lung disease, *COPD* chronic obstructive pulmonary disease, *ARDS* acute respiratory distress syndrome

There were no difference between low- and high-volume centers in other clinical outcomes including ICU and hospital length of stay (Table 4).

**Table 4:** Outcomes according to the case-volume

		Case-volume (LTs per year)^a^		
	Total	Low volume centers (< 5 LTs/year)	High volume centers (≥ 5 LTs/year)	*P* value
ICU length of stay, days	36.5 ± 69.4	38.7 ± 31.1	35.9 ± 77.1	0.764
Hospital length of stay, days	82.1 ± 88.6	91.8 ± 74.8	79.2 ± 92.1	0.292

Data are presented as mean ± SD. *LT* lung transplantation, *ICU* intensive care unit

^a^Average number of lung transplantations from 2011 to 2016

## Discussion

In this study, we analysed patients who underwent LT in the past ten years in Korea and found no definite association between case-volume and in-hospital mortality or long-term survival.

The association between higher case-volume and superior outcomes has been repeatedly demonstrated in various surgical procedures [5–9]. This relationship becomes especially important for high-risk procedures such as solid organ transplantation since the number of available organs are limited [10, 11, 15, 20, 21]. The effect of case-volume in LT on post-LT survival have been reported since the 2000s [2, 12–15], with the most recent study analysing 13,506 adult LT recipients suing United Network for Organ Sharing data from 2005 through 2013. There was a trend towards higher one- and five-year survival after in higher case-volume (≥ 30 LTs per year) centers [2].

However, our study results were not in accord with previous reports on case-volume and patient outcome after LT. There are a few differences compared to previous studies that may provide some explanations. The number of recipient we analysed was only 315 in seven centers with volume categories constructed with five LTs per year. There were three centers in high-volume centers including one center with a case-volume of 25 LTs per year, but the other two centers had less than ten. In studies which included more than 10,000 LT recipients from more than 70 centers for analysis, 20–30 LTs per year was the cut-off to differentiate low- and high-volume centers [2, 12, 13]. Therefore, the total number of LT patients and thus the cut-off for low- and high-volume centers may have been too small to show the effect of case-volume on the patient outcomes. Nevertheless, the trend towards better long-term survival after LT in high volume centers may suggest a potentially positive case-volume effect for regions/countries in early stages of LT.

In addition to the number of patients, the distribution of underlying lung disease was also different. The proportion of LT recipients diagnosed with IPF was higher in our study compared to previous studies. ISHLT reported COPD as the most common lung disease in patients receiving LT (36%), followed by interstitial lung disease (including IPF) (30%) [3]. In our study, the proportion of recipients with ILD or IPF was more than 60% of the all LT patients and increased with higher case-volume. Although IPF is known to be associated with higher mortality after LT compared to other pulmonary diseases calling for LT [22], our result showed higher mortality in COPD patients after LT. This may be related to the relative shortage of lungs for transplantation and higher mortality of patients on the waiting list in Korea compared to other countries [23, 24].

Although experience seems to be the logical reason for the association between surgical case-volume and patient outcomes in complex surgical procedures, the precise mechanism is unclear. Centers with different case-volume may differ in the process and/or quality of perioperative management. Prior studies in esophagectomy and pediatric heart surgery showed that implementation of standardized clinical care pathway for better perioperative care, rather than the case-volume of individual surgeon, improved survival [25–28]. In addition, a review of more than 12,000 patients with primary LT showed no difference between low- (< 21.8 LTs per year) and high-volume centers (> 34.2 LTs per year) with regard to postoperative complications. However, high-volume centers were best able to minimize the adverse effects of postoperative complications leading to improved short- and long-term survival [12]. The number of intensivists [29–31], presence of multidisciplinary teams, and standardized clinical pathway for LT may have influenced perioperative care of LT [2, 30]. Our findings may also have been influenced by the overall perioperative management of the LT patient rather than case-volume itself.

A minimum case-volume threshold or criteria may be required for optimal outcome and survival. The Centers for Medicare and Medicaid certification in the US adopted the concept of minimum volume requirements for LT with a volume threshold of ten LTs per year [32]. However, case-volume may be an imperfect surrogate of performance and not all low-volume centers have poor survival rates as in our study [2, 33, 34]. Studies using the National Surgical Quality Improvement data, which includes prospectively monitored with risk-adjusted 30-day mortality and morbidity, failed to demonstrate a correlation between case-volume and outcomes of various surgical procedures [35, 36]. The emphasis was on the systems of care rather than case-volume. They advocated against the use of volume criterion as a surrogate measure of surgical outcome emphasizing the systems of care are more important than the volume in determining overall quality [36]. Our results also showed no difference between low- and high-volume centers regarding risk-adjusted short- and long-term survival. However, the relatively poor outcome of the institution with the highest case-volume seems to have influenced our results. Detailed clinical assessment of patients who received LT in the highest case-volume center may be required to understand the reason for the relatively poor outcome. Further research and discussion will be necessary to establish standards that would be most beneficial to LT patients in Korea.

Korea has a single-payer government regulated healthcare system. The National Health Insurance Service in Korea is the single-payer programme which mandatorily covers all residents in Korea [16, 17]. The data in our analysis is from database of the Health Insurance Review and Assessment Service which regulates healthcare quality, evaluates healthcare performance, and reviews reimbursement and claims [17]. Thus, it represents the entire Korean population and is often used as a population-based database [17]. One of the strengths of our study is that all LTs performed in Korea during the study period was included.

There are several shortcomings of our study. First, the analysis is limited by the retrospective use of administrative data. The inclusion of all patients who underwent LT in Korea provides a strong explanatory power and a complete picture of the situation in one country. Second, only hard outcome measures such as in-hospital mortality was analysed. Despite adjustment using the Elixhauser comorbidity index, not all potential confounders including patients’ individual clinical features and surgeon variables were accounted for. Although a better understanding of the relationship between case-volume and survival rate after LT may have been provided with the addition of clinical data, the limitations of administrative data in this aspect was not amendable. Finally, the threshold for case-volume categories were arbitrarily constructed with only seven LT centers included. Moreover, most of the cases were concentrated in one highest volume center with a single LT surgeon. The uneven distribution of LT patients within a small number of LT centers introduces a higher chance of surgeon specific factors rather than case-volume effect of LT.

## Conclusions

In conclusion, we were unable to demonstrate an association between case-volume of LT and short- and long-term mortality in Korea. Although our study showed a trend towards improved results in higher volume centers, it seems possible to have favorable outcomes in centers with an annual LT case volume as low as 5. Although a further study with more data would be required for a more definitive conclusion, our results may be used as a platform for a discussion on optimal organ allocation for transplantation and improve patient survival after LT.

## Abbreviations

ARDS: Acute respiratory distress syndrome
CI: Confidence interval
COPD: Chronic obstructive pulmonary disease
HR: Hazard ratio
ICU: Intensive care unit
ILD: Interstitial lung disease
IPF: Idiopathic pulmonary fibrosis
ISHLT: International Society for Heart and Lung Transplantation
LOS: Length of stay
LT: Lung transplantation
NHI: National Health Insurance
NHIS: National Health Insurance Service
OR: Odds ratio
